# Chlorogenic Acid Targets Cell Integrity and Virulence to Combat *Vibrio parahaemolyticus*

**DOI:** 10.3390/foods14193416

**Published:** 2025-10-03

**Authors:** Huan Liu, Jie Zhao, Yile Shi, Juanjuan Cao, Yanni Zhao

**Affiliations:** 1 School of Food Science and Engineering, Shaanxi University of Science & Technology, No. 6 Xuefu Road, Xi’an 710021, China; liuhuan@sust.edu.cn (H.L.); 18435128287@163.com (J.Z.); 15229761690@163.com (Y.S.); 19829264920@163.com (J.C.); 2 Shaanxi Research Institute of Agriculture Products Processing Technology, No. 6 Xuefu Road, Xi’an 710021, China

**Keywords:** chlorogenic acid, *Vibrio parahaemolyticus*, antibacterial mechanism, attenuated effect

## Abstract

*Vibrio parahaemolyticus* is a primary foodborne pathogen in seafood that endangers consumers’ health. It is vital to develop novel prevention and control strategies due to its extensive transmission and drug resistance. This work aimed to examine the antibacterial and anti-virulence efficiency of chlorogenic acid (CA) against *V. parahaemolyticus*. The minimum inhibitory concentration (MIC) of CA is 6 mg/mL. CA realized its antibacterial effect by damaging the cell wall and membrane, evidenced by the leakage of alkaline phosphatase, intracellular proteins and nucleic acids, potassium ion, and glucose, the increasing malondialdehyde and reactive oxygen species, as well as morphological observations under scanning and transmission microscopes and live and dead cell observations under laser confocal microscopy. When *V. parahaemolyticus* was treated with CA at sub-inhibitory doses, its hydrophobicity, extracellular polysaccharide synthesis, motility, and biofilm formation were all significantly inhibited. Moreover, CA effectively protected salmon from the contamination of *V. parahaemolyticus* with a prolonged shelf life. These findings indicate that CA possesses antibacterial activity against *V. parahaemolyticus*, suggesting its potential value for controlling *V. parahaemolyticus*-associated seafood infections.

## 1. Introduction

*Vibrio parahaemolyticus*, a rapidly reproducing foodborne pathogen [1,2], is prevalent in seafood, including fish, shrimp, oysters, crabs, and shellfish [3,4]. Infection with this bacterium causes symptoms ranging from abdominal pain and vomiting to severe outcomes like shock and even death [5]. It has emerged as a prominent cause of foodborne illness outbreaks throughout the world over recent years [3]. With the developing global warming concern, an increasing number of food poisoning cases caused by *V. parahaemolyticus* have been reported in South Korea, the United States, Australia, and southern coastal China, posing serious health dangers [6]. Between 2001 and 2021, 71 seafood disease outbreaks associated with *V. parahaemolyticus* were reported, and these outbreaks led to 994 illnesses [7]. National-based prospective surveillance of all-age patients with acute diarrhea conducted in China from 2009 to 2018 demonstrated that *V. parahaemolyticus* ranks as the fourth most prevalent bacterial cause of diarrhea, trailing only diarrheagenic *Escherichia coli*, nontyphoidal *Salmonella*, and *Shigella* [8]. *V. parahaemolyticus* RIMD2210633, an O3:K6 serotype strain, was firstly isolated at the Kansai International Airport quarantine station in 1996 from a patient with travelers’ diarrhea and the whole genome sequencing was completed in 2003 [9,10].

Antibiotics served as vital agents in combating bacterial infections [11]. Nevertheless, the overuse and misuse of antibiotics has led to the emergence of antibiotic-resistant bacteria [12], threatening both food safety and public health. The harsh current situation makes the creation of novel antibacterial agents an urgent concern that must be tackled. Chlorogenic acid (CA) is a phenolic acid derived from caffeic acid and quinic acid [13], and is a secondary metabolite produced by various plants through the shikimic acid pathway [14]. CA, known as “plant gold,” exhibits a variety of biological activities, including antibacterial [15], antioxidant [16], anti-inflammatory [17], anti-tumor [18], and hepatoprotective effects [19]. CA acts as a multifaceted antimicrobial agent by antagonizing biofilm formation, destroying the cell membrane and increasing the membrane permeability and offers promise for combating *Yersinia enterocolitica* infections and contamination in milk [20]. CA’s minimum inhibitory concentration against *Streptococcus pyogenes* was 500 μg/mL, and it works perhaps by ribosomal subunit downregulation, interference with lipid metabolism, and intracellular reactive oxygen species (ROS) scavenging [21]. Moreover, CA displayed good antimicrobial potential against *Aeromonas hydrophila* (MIC = 6.4 mg/mL) and repressed the expression of genes involved in quorum sensing, biofilm formation, and hemolysin [22]. In mice with colitis, CA inhibited the growth of the harmful bacteria including *Bacteroides*, *E. coli*, and *Shigella*, but promoted the population of beneficial bacteria [17]. So far, research on CA’s antibacterial activity against *V. parahaemolyticus* has yet to be fully understood.

In this work, we evaluated the antibacterial and anti-virulence effects of CA against *V. parahaemolyticus* RIMD2210633. In addition, the potential antibacterial mechanisms and its protective efficacy against *V. parahaemolyticus* infection in salmon were characterized. Our findings will provide a preliminary theoretical foundation for the control of *V. parahaemolyticus* in aquaculture and seafood preservation.

## 2. Materials and Methods

### 2.1. Bacterial Strains and Cultivation

The *V. parahaemolyticus* RIMD2210633 was stored in Luria–Bertani (LB) at −80 °C containing 20% (*v*/*v*) glycerol [23]. A primary bacterial solution was prepared by inoculating 5 mL of LB culture with 1% (*v*/*v*) of *V. parahaemolyticus* and then cultivated for 12 h at 37 °C at 200 rpm. The concentration of the primary bacterial suspension was determined by measuring its optical density at 600 nm (OD_600_) using a ultramicro UV-Vis spectrophotometer (Q6000+, Quawell, San Jose, CA, USA). The primary bacterial suspension was then adjusted to the desired working concentration of OD_600 nm_ = 1.0 (approximately 1.0 × 10^6^ CFU/mL) for subsequent experiments.

### 2.2. Minimum Inhibitory Concentration and Growth Curve Determination

The minimum inhibitory concentration (MIC) was determined by the 2-fold dilution method described by Cao et al. [24]. For growth curve detection, the primary bacterial solution was added into the fresh LB broth supplemented with different concentrations (0, 1/8 MIC, 1/4 MIC, 1/2 MIC, MIC, 2 MIC) of chlorogenic acid (CA) (Yuanye, Shanghai, China) with a 1% inoculation amount and then cultivated at 37 °C and 200 rpm, respectively. CA-free LB broth served as a control. The optical density at 600 nm (OD_600_) of samples taken every hour was recorded.

### 2.3. Measurement of Extracellular Nucleic Acid and Protein Content

The primary bacterial solution was inoculated into the fresh LB broth and cultured for 3 h at 37 °C and 200 rpm. Then CA was added into the cultures to reach the final concentrations of 0, 1/4 MIC, 1/2 MIC, and MIC, respectively. Bacterial solutions were grown at 37 °C for 6 h. After centrifugation (8000 rpm, 3 min), the absorbance of the supernatants at 260 nm and 280 nm was measured using an ultramicro UV spectrophotometer (Q6000+, Quawell, San Jose, CA, USA).

### 2.4. Measurement of Extracellular Potassium Ion and Glucose Content

The bacterial samples were generated as indicated in Section 2.3, and the extracellular potassium ion and glucose contents were determined using a potassium ion assay kit (Jiancheng, Nanjing, China) and a glucose assay kit (Jiancheng, Nanjing, China) following the manufacturer’s instructions, respectively.

### 2.5. Determination of Malondialdehyde (MDA) Content

After the treatment with CA at different concentrations as described in Section 2.3, and the MDA content in the supernatant was determined using an MDA assay kit (Jiancheng, Nanjing, China) according to the manufacturer’s instructions. After thoroughly mixing the sample, TBA reagent, and acidic buffer, the mixture was incubated in a water bath at 95 °C for 30 min. After cooling, the supernatant was collected, and its absorbance at 532 nm was recorded.

### 2.6. Determination of Intracellular Reactive Oxygen Species (ROS) Content

The intracellular ROS content of *V. parahaemolyticus* treated with CA at different concentrations were detected by the method proposed by Liao et al. [25] with slight modifications. Bacteria were treated and collected as outlined in Section 2.3. After removing the supernatant by centrifugation, 200 µL of 2,7-Dichlorodihydrofluorescein diacetate (10 µmol/L) solution was added, and the mixtures were incubated at 37 °C for 30 min. Then the bacterial cells were washed three times with PBS buffer. The fluorescence intensity of the bacterial suspension was measured using the excitation wavelength of 488 nm and the emission wavelength of 525 nm.

### 2.7. Determination of Alkaline Phosphatase Content

Bacterial solutions were prepared following the protocol in Section 2.3, and the alkaline phosphatase (AKP) concentration in the supernatant was determined with an AKP assay kit according to the manufacturer’s instruction (Jiancheng, Nanjing, China).

### 2.8. Staining of Live and Dead Cells

Bacterial solutions were prepared following the same procedure described in Section 2.3, cells were collected by centrifugation at 8000 rpm for 5 min and washed with PBS. Then the bacterial cells were suspended with 1 mL of 10 µL/mL Green Fluorescent Nucleic Acid Stain (SYTO-9) (KeyGEN, Nanjing, China) and stained for 15 min at 37 °C in the dark. Consequently, 10 µL of propidium iodide (PI) staining solution (Beyotime, Shanghai, China) was added and then stained for another 15 min at 37 °C in the dark. Then the cell was observed by confocal laser scanning microscopy (Carl Zeiss, Oberkochen, Germany).

### 2.9. Observation of Cell Morphology Using Field Emission Scanning Electron Microscopy (FESEM) and Transmission Electron Microscopy (TEM)

As described in 2.3, the bacterial cells were collected and suspended in 1 mL of a 2.5% (*v*/*v*) glutaraldehyde solution. Then the cells were fixed for 4 h at 4 °C, centrifuged for 3 min at 8000 rpm, and then rinsed three times with 1 mL of PBS. Subsequently, the cells were dehydrated in steps to 1 mL of ethanol at varying concentrations (30%, 50%, 70%, 90%, 95%, and 100% (*v*/*v*)) twice. After the spray gold treatment, the cell morphology was observed by a scanning electron microscope (Sigma 300, Carl Zeiss, Oberkochen, Germany).

For TEM observation, the dehydrated cells were prepared according to the procedures of the SEM assay and counterstained with 1% phosphotungstic acid (*v*/*v*). The ultrastructural morphology of the cell was observed by a transmission electron microscope (TECNAI G2F20, FEI, Hillsboro, OR, USA).

### 2.10. Determination of Hydrophobicity

The hydrophobicity of *V. parahaemolyticus* treated with CA at different concentrations were evaluated by the method described by Lebeloane et al. [26] with some modifications. In brief, the primary bacterial solution was inoculated into fresh LB broth with CA at concentrations of 1/8 MIC, 1/4 MIC, and 1/2 MIC, respectively, and cultivated for 9 h at 37 °C and 200 rpm. Then 1 mL of bacterial cultures and an equivalent volume of toluene were vortexed to mix well. After settling at room temperature and layering, the absorbance of the aqueous phase layer at 600 nm was recorded. The hydrophobicity was calculated using the following formula:

Hydrophobicity = 1 − (OD_600 nm_ of water phase after vortexing/initial OD_600 nm_ before vortex formation) × 100%

### 2.11. Determination of Extracellular Polysaccharides

The bacterial cultures treated with CA at different concentrations were prepared as described in Section 2.10. The supernatant (1 mL) harvested by centrifugation at 8000 rpm for 5 min was mixed with 3 mL of 95% (*v*/*v*) ethanol solution and placed at 4 °C for precipitation. The sediment was added with 1 mL of water, 1 mL of 5% (*v*/*v*) phenol, and 5 mL of concentrated sulfuric acid in turn and mixed well. The solution’s OD_490 nm_ value was measured after 20 min.

### 2.12. Determination of Biofilm

The primary bacterial solution was inoculated into fresh LB broth with CA at concentrations of 0, 1/8 MIC, 1/4 MIC, and 1/2 MIC, respectively, and cultured for 24 h. Following the removal of the bacterial solution, the biofilm was stained with crystal violet and then dissolved in 1 mL of 33% (*v*/*v*) acetic acid. The absorbance of the solution at 570 nm was determined.

### 2.13. Motility Assay

For the swarming assay, an aliquot of 5 µL primary bacterial solution was spotted onto the LB plates (1.5% (*w*/*v*) agar) supplemented with CA at various concentrations (0, 1/4 MIC, 1/2 MIC, MIC), respectively. While for swimming detection, an aliquot of 2 µL primary bacterial solution was spotted onto the LB plates (0.3% (*w*/*v*) agar) added with CA at various concentrations (0, 1/4 MIC, 1/2 MIC, MIC), respectively. The plates were incubated at 37 °C for 24 h and the motility was photographed.

### 2.14. Application in Salmon Preservation

The protection effect of CA on salmon against *V. parahaemolyticus* was assessed according to the method utilized by Luo et al. [27] with some modifications. Briefly, the fresh salmon purchased from the local market (Xi’an, Shaanxi, China) was washed twice with sterile water and irradiated with ultraviolet light for 30 min. The salmon (25 g) was mashed into a homogenate and mixed well with 250 μL the bacterial suspensions (OD_600 nm_ = 1.0). Afterwards, CA at different concentrations of 0, MIC, 2 MIC, 4 MIC, and 8 MIC was added to the salmon and mixed well, respectively. After incubation at 4 °C, samples (1 g) in triplicate were collected at 0, 2, 4, 6, and 8 days from each group, followed by dilution with PBS, and finally counted with TCBS plates.

### 2.15. Statistical Analysis

GraphPad Prism 9.0.0 was utilized for the analysis and plotting of all the data. All experiments were performed in triplicate at least, and data were presented as mean ± standard deviation. The significance of differences between the control group (designated as “0” in analysis) and treatment groups was assessed using a one-way ANOVA followed by Dunnett’s post hoc test. *p* < 0.05 was regarded as significant, and *p* < 0.01 as extremely significant, denoted by * and **, respectively.

## 3. Results and Discussion

### 3.1. The Effect of Chlorogenic Acid on the Growth of V. parahaemolyticus

**Figure 1:**
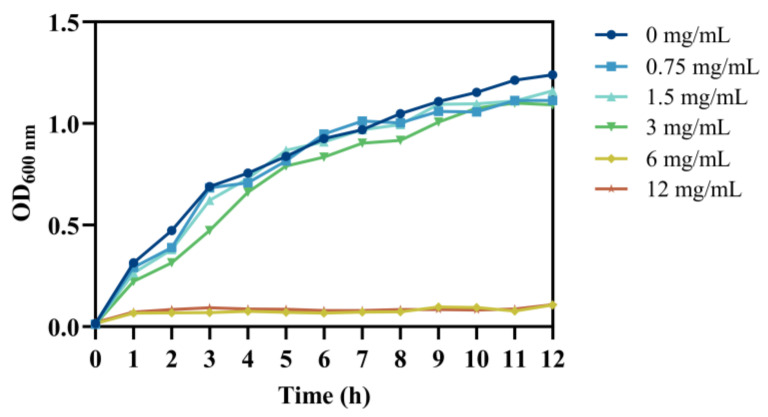
Growth curve of *V. parahaemolyticus* treated with chlorogenic acid at different concentrations.

The minimum inhibitory concentration (MIC) of chlorogenic acid (CA) was determined to be 6 mg/mL by the double-dilution method. Further, CA’s effect on the growth of *V. parahaemolyticus* RIMD2210633 was shown in Figure 1. In the control group without CA, *V. parahaemolyticus* exhibited rapid proliferation and entered the exponential growth phase with a negligible lag phase. In the group treated with 3 mg/mL CA, *V. parahaemolyticus* cultures showed lower bacterial concentrations throughout the detection period compared to the control. In the presence of CA with a concentration of 6 mg/mL or higher, no detectable growth of *V. parahaemolyticus* occurred. The results of the growth curve fully indicate that the MIC of chlorogenic acid against *V. parahaemolyticus* RIMD2210633 was 6 mg/mL. Due to genetic diversity, the susceptibility and precise mechanisms of different strains to CA may vary, especially between clinical and environmental isolates. A further research including a wider range of clinical and environmental isolates should be conducted to fully evaluate the anti-*V. parahaemolyticus* activity of CA.

### 3.2. CA Targeted on the Cell Membrane and Cell Wall of V. parahaemolyticus

#### 3.2.1. The Effect of CA on the Extracellular Nucleic Acid and Protein Content of *V. parahaemolyticus*

**Figure 2:**
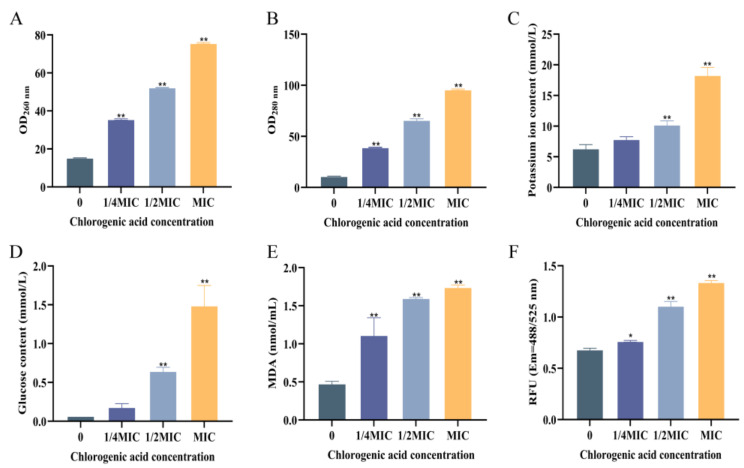
Effect of chlorogenic acid at different concentrations on the cell membrane of *V. parahaemolyticus*. (**A**) The content of extracellular nucleic acids, (**B**) extracellular protein content, (**C**) extracellular potassium ion content, (**D**) extracellular glucose content, (**E**) MDA, and (**F**) intracellular reactive oxygen species content. Each value represents the average of three independent measurements. The bar represents the standard deviation (n = 3), * *p* < 0.05, ** *p* < 0.01.

The cell membrane is an important component for maintaining normal cell growth and metabolism. If the cell membrane is damaged, it will cause the leakage of biological macromolecules such as nucleic acids and proteins inside the cell. As shown in Figure 2A,B, the contents of both extracellular DNA and proteins of *V. parahaemolyticus* were low in the control group without CA, indicating the integrity of the cell. For the groups treated with CA, the levels of both DNA and proteins outside the cell were dramatically increased in contrast to the control, and this phenomenon demonstrated that CA caused the cell membrane damage. The leakage of the intracellular macromolecules increased along with the rising CA concentration. Similarly, CA at MIC caused significant protein and DNA leakage in *Shigella dysenteriae* [28]. Moreover, CA displayed good antibacterial activity against *Pseudomonas aeruginosa* with an MIC of 5 mg/mL and caused the leakage of intracellular substances, including the protein and ATP. CA treatment resulted in the reduction in LPS contents and also the expression of genes involved in LPS synthesis, such as LPxB and LPxC for lipid A biosynthesis, with downregulated levels by 33.6 and 15.1 times compared to control groups [29].

#### 3.2.2. CA’s Action on the Extracellular Potassium Ion and Glucose Content of *V. parahaemolyticus*

When cells are growing normally, potassium ions and glucose are typically transferred from the extracellular environment into the intracellular space by active transport to fulfill the requirements for cellular survival. Once the cell membrane is damaged and becomes more permeable under the harsh external environment, potassium ions and glucose will flow out of the cell and into the outside space. The contents of extracellular potassium ions and glucose in *V. parahaemolyticus* culture treated with CA at various concentrations were displayed in Figure 2C,D. In the control, both the extracellular potassium ions and glucose contents were low, indicating the integrity of the cell membrane. No substantial difference existed between the 1/4 MIC group and the control group. However, the extracellular potassium ions and glucose increased obviously compared to the control group, respectively, when CA concentration reached 1/2 MIC or MIC. For the MIC treatment group, the extracellular potassium ion concentration reached 18.31 mmol/L, and the glucose concentration reached 1.49 mmol/L. High concentrations of CA treatment resulted in significant damage to the cell membrane of *V. parahaemolyticus*, thus increasing its permeability and causing the leakage of small molecular substances.

#### 3.2.3. The Effect of CA on Lipid Oxidation of *V. parahaemolyticus*

The change in MDA content can reflect the degree of lipid peroxidation damage on the cell membrane. As shown in Figure 2E, the MDA content in the control group was very low (0.48 nmol/mL) in the absence of CA. Upon the addition of CA at varying concentrations (1/4 MIC, 1/2 MIC, and MIC), the MDA content increased significantly in the treatment groups, and a positive correlation between MDA content and CA concentration was observed. For the group with CA at MIC, the MDA content reached 1.74 nmol/mL. It can be seen that CA can cause oxidative damage to the cell membrane of *V. parahaemolyticus*, and the degree of this damage increased with the drug concentration. *Ailanthus altissima* extract, with CA as the second-highest content component, was discovered to cause an increase in both MDA and reactive oxygen species content, thus resulting in the lipid peroxidation and the integrity damage of cell membranes in *S. aureus* and *E. coli* [30].

#### 3.2.4. The Effect of CA on the Intracellular Reactive Oxygen Species Content of *V. parahaemolyticus*

Reactive oxygen species (ROS) are products of normal oxygen metabolism in cells, which work together with antioxidant enzymes to maintain intracellular oxygen balance metabolism. When cells are stimulated by external factors, the activity of antioxidant enzymes decreases, and the intracellular oxygen balance is disrupted, resulting in the accumulation of a large amount of ROS. Abnormal ROS can lead to lipid oxidation, protein oxidation, DNA strand breakage, and base modification [31]. The CA’s action on the intracellular ROS content of *V. parahaemolyticus* was shown in Figure 2F. After treatment with CA at 1/4 MIC, 1/2 MIC, and MIC, the intracellular ROS content of *V. parahaemolyticus* increased significantly in pace with the CA concentration in contrast to the control group. For the group with the addition of CA at MIC, the intracellular ROS content increased by 97.06% compared to the control. This indicated that CA can break the intracellular oxygen balance metabolism, causing the accumulation of ROS and abnormal cellular metabolism in *V. parahaemolyticus*. In contrast, CA may exert its antibacterial action by decreasing ribosome synthesis, affecting lipid metabolism, and scavenging intracellular ROS by higher expression of oxidation-reduction-related proteins in *S. pyogenes* [21]. CA substantially reduces *Fusarium oxysporum*’s conidia germination, embryonic tube elongation, cell survival, and hyphal development. It can also increase the production of ROS, which causes *F. oxysporum* cells to undergo apoptosis [32].

#### 3.2.5. The Effect of Chlorogenic Acid on Alkaline Phosphatase of *V. parahaemolyticus*

**Figure 3:**
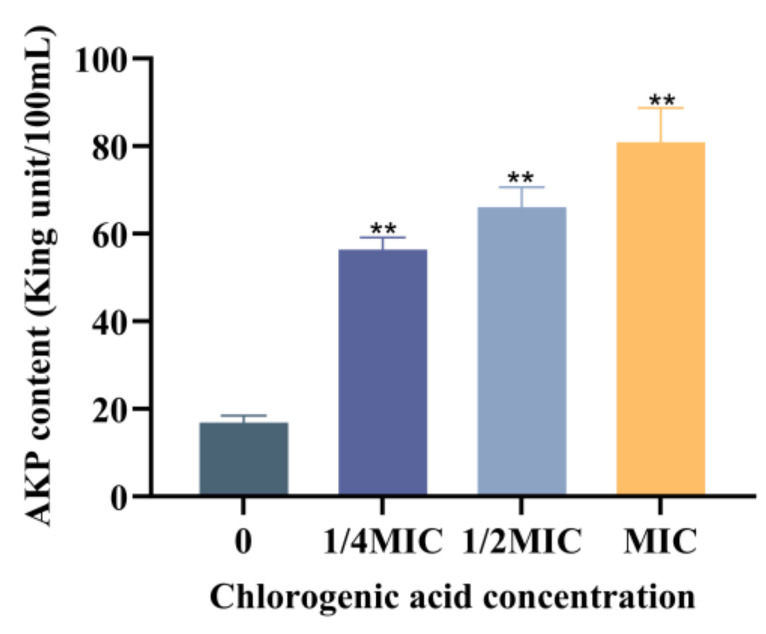
Effect of chlorogenic acid at different concentrations on extracellular alkaline phosphatase content of *V. parahaemolyticus.* Each value represents the average of three independent measurements. The bar represents the standard deviation (n = 3), ** *p* < 0.01.

Alkaline phosphatase, located between the bacterial cell wall and membrane, cannot be detected outside the cell during normal cell survival. However, when the cell wall is damaged, alkaline phosphatase leaks out of the cell. The effect of CA on the integrity of *V. parahaemolyticus* cell wall was studied by measuring the content of extracellular alkaline phosphatase. As shown in Figure 3, the level of extracellular AKP of *V. parahaemolyticus* was low in the control. When *V. parahaemolyticus* was treated with CA at concentrations of 1/4 MIC, 1/2 MIC, and MIC, respectively, the extracellular AKP contents in all the groups were significantly elevated compared to the control. For the group treated with CA at MIC, the extracellular AKP content reached 81.39 King units/100 mL. This indicated that CA can destroy the cell wall integrity of *V. parahaemolyticus*. CA also inhibited the growth of *Salmonella typhimurium* SL1344 by targeting and doing damage to the bacterial cell wall [33].

#### 3.2.6. Staining of Live and Dead Cells

**Figure 4:**
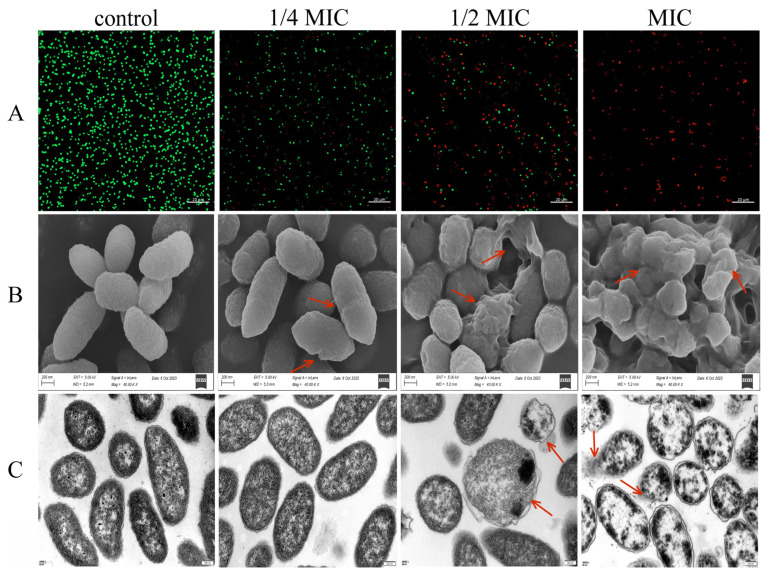
Effect of chlorogenic acid at different concentrations on *V. parahaemolyticus* were observed through confocal laser scanning microscopy (**A**), field emission scanning electron microscopy images (**B**), and transmission electron microscopy images (**C**). Green fluorescence represents live cells, while red fluorescence represents dead cells. The red arrows indicate wrinkled surfaces and cell collapse.

Propidium iodide (PI) is a membrane-impermeant dye. In live cells with intact plasma membranes, PI is prevented from entering the cell. While in cells with compromised membrane integrity or increased permeability, PI freely enters the cell, binds to DNA, and causes the cell to emit red fluorescence. SYTO9 dyes are membrane-permeant, can enter live cells to bind nucleic acids, and emit green fluorescence [34]. The staining of live and dead cells treated with CA at various concentrations was observed by CLSM, and the result was shown in Figure 4A. For the control group without the addition of CA, the cells were stained by SYTO9 and had strong green fluorescence, and almost no red fluorescence was detected, which means that the cells are alive and intact. With the addition of CA, the proportion of PI-labeled cells was increased along with the drug level. In the MIC-treated group, almost all the cells were stained by PI and exhibited red fluorescence, which indicates that CA caused cell membrane damage and increased permeability.

#### 3.2.7. CA’s Effect on the Cell Morphology and Ultrastructure of *V. parahaemolyticus*

SEM was used to examine the morphological alterations of bacterial cells in order to better understand CA’s impacts on *V. parahaemolyticus*, and the result was shown in Figure 4B. Untreated *V. parahaemolyticus* cells in the control were short rod-shaped, with a smooth and intact bacterial surface. After treatment with CA at a dose of 1/4 MIC, most of the cells displayed typical *Vibrio* morphology. After treatment with chlorogenic acid at a concentration of 1/2 MIC, some bacteria cells displayed obvious shrinkage, surface roughness, and even rupture. After being treated with CA at MIC, the *V. parahaemolyticus* cells underwent severe wilting, invagination, deformation, and even emerged without entire cell morphology.

In further, the CA’s action on the ultrastructure of *V. parahaemolyticus* was determined by transmission electron microscopy. As shown in Figure 4C, the bacterial cells without CA treatment exhibited an intact cell structure, cell membranes were in intimate contact with the cell wall, and the cytoplasm was homogenous. After treatment with CA at 1/4 MIC, no notable variation in cell morphology was seen as compared to the control group. While in the CA treatment group with higher levels (1/2 MIC or MIC), huge vacuoles emerged within the cells, the cytoplasm diminished and irregularly distributed, and the bacterial cell wall and membrane deteriorated, causing the leakage of cell contents. The extent of cellular damage escalated with rising CA concentration.

At present, the CA’s antibacterial mechanism is mainly manifested in two aspects [30]: (1) Change in the permeability of the cell membrane [35]. CA is a highly polar substance that easily binds to lipids on the cell membrane, thereby altering the structure of the cell membrane and increasing its permeability, leading to molecular leakage within the cell and promoting bacterial death [36]. Sun [37] found that CA can alter the permeability of the inner and outer membranes of *Salmonella*, causing the leakage of intracellular proteins and ATP, ultimately leading to bacterial death. Martine [38] found that CA displayed a significant inhibitory effect on the growth of *Sclerotinia sclerotiorum*, *Verticillium dahliae*, *Botrytis cinerea*, and *Fungi imperficti*, and can inhibit their growth by suppressing the permeability of fungal spore membranes in the early stage. (2) Influence on the intracellular ROS content [30]. Under normal circumstances, the production and consumption of ROS within the bacterial cells maintain a dynamic balance. If the content of ROS is too high, it can lead to the oxidation of proteins, nucleic acids, and lipids inside the cell [39], resulting in intracellular damage. If ROS is cleared, it may have antibacterial effects [21]. Lee et al. [40] found that CA-induced apoptosis in *E. coli* is not due to the production of ROS, but rather due to the depletion of intracellular ROS by CA. The depletion of ROS may affect bacterial intracellular signaling pathways, leading to cell death.

### 3.3. The Effect of Chlorogenic Acid on the Attenuation of V. parahaemolyticus

#### 3.3.1. The Effect of Chlorogenic Acid on the Hydrophobicity of *V. parahaemolyticus*

**Figure 5:**
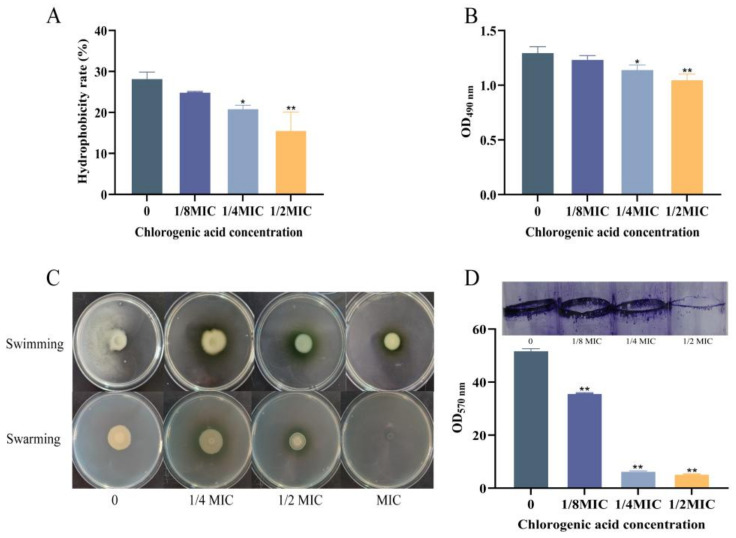
Effect of chlorogenic acid at different concentrations on virulence factors of *V. parahaemolyticus*. (**A**) Hydrophobicity, (**B**) Extracellular polysaccharide content, (**C**) Motility assay, (**D**) Biofilm formation. Each value represents the average of three independent measurements. The bar represents the standard deviation (n = 3), * *p* < 0.05, ** *p* < 0.01.

Hydrophobicity is one of the key factors for cell adhesion [2] and biofilm formation [41]. As shown in Figure 5A, the hydrophobicity of *V. parahaemolyticus* treated with CA at 1/4 MIC or 1/2 MIC decreased significantly compared to the control group, respectively. The hydrophobicity was repressed along with the increasing concentrations of CA, with the lowest level of 15.68% in the 1/2 MIC group. This indicated that CA can effectively reduce the hydrophobicity of *V. parahaemolyticus*, thus impairing the adhesion ability and biofilm formation of *V. parahaemolyticus*. According to a study, CA greatly influences and increases the hydrophobicity of *S. aureus*’s surface in a dose-dependent manner, producing antibacterial effects [42].

#### 3.3.2. The Effect of CA on the Extracellular Polysaccharide Content of *V. parahaemolyticus*

Extracellular polysaccharides (EPSs) are essential components of the extracellular matrix in biofilm production. These matrix components fill the gaps between bacterial cells and aggregate cells in biofilm to protect them from toxic substances or other stress factors in the external environment [43]. As shown in Figure 5B, the EPS content of *V. parahaemolyticus* in the group containing CA at 1/8 MIC displayed no significant difference compared to the control group. While for the groups with higher concentrations of CA (1/4 MIC or 1/2 MIC), the EPS production by *V. parahaemolyticus* was dramatically repressed in contrast to the control.

#### 3.3.3. The Effect of CA on the Motility of *V. parahaemolyticus*

*V. parahaemolyticus* has a polar flagellum for swimming in liquids and multiple lateral flagella for surface swarming [44]. In order to form a biofilm, bacterial cells use the polar flagella system and lateral flagella system to move, attach, and aggregate on the host surface [45]. Effects of different concentrations of CA on the motility of *V. parahaemolyticus* can be seen in Figure 5C. Cells without CA exhibited good diffusion capacity on the culture medium surface, which indicated that they swim and swarm well. However, the cells treated with different concentrations of CA exhibited notable size on the culture medium surface compared to the control group. This suggested a gradual decrease in swimming and swarming distances of *V. parahaemolyticus* on soft and hard surfaces with increasing CA concentration. This indicated that CA at sub-inhibitory concentrations repressed the motility of *V. parahaemolyticus* in a drug-level-dependent manner.

#### 3.3.4. The Effect of CA on Biofilm Formation of *V. parahaemolyticus*

Biofilms are structurally complex microbial assemblies that adhere to biological or non-biological surfaces [46]. *V. parahaemolyticus* in biofilms has stronger resistance to antibiotics and disinfectants than that in planktonic condition [47]. Biofilms can help microorganisms resist adverse environmental factors and host immune system clearance. As shown in Figure 5D, in the control group without CA, *V. parahaemolyticus* adhered to the solid surface and formed a dense biofilm. However, in the treatment groups with CA at concentrations of 1/8 MIC, 1/4 MIC, and 1/2 MIC, the biofilm formation was significantly repressed, and the biofilm content was reduced by 30.97%, 87.48%, and 89.69% compared to the control group, respectively. The findings indicate that CA can reduce biofilm formation in a dose-dependent manner, thereby weakening the virulence of *V. parahaemolyticus*.

The process of biofilm formation is generally believed to have five stages: The first stage is the reversible adhesion stage of planktonic bacteria, the second stage is the irreversible adhesion stage, and the degree of bacterial colonization depends on the roughness and hydrophobicity of the substrate, as well as the composition of the cell surface [48], the third stage is the formation of extracellular polymer matrix, the fourth stage is the maturation stage of biofilms, and the fifth stage is the stage of biofilm degradation and detachment [49], where extracellular polymers begin to degrade and some bacteria detach from the biofilm into the environment [50]. *V. parahaemolyticus* interacts with the attached surface through flagella, pili, and secreted adhesins, leading to the formation of biofilms. After successful adhesion, it promotes surface colonization by reducing flagella movement, adhesion protein secretion, and extracellular polysaccharide synthesis [51].

CA exhibited a good antibiofilm potential against *P. putida*, and it inhibited the biofilm formation of *P. putida* via suppressing biofilm maturation, changing the microstructure and metabolites of the biofilm, and weakening forces of biofilms as well as interfering with the elements involved in the quorum sensing systems, such as Las, RhL, and Pqs [52]. Chlorogenic acid-grafted-chitosan (CS-g-CA) at sub-concentrations inhibited the biofilm formation by decreasing the content of extracellular polymers, weakening the motility and adhesion of *P. fluorescens*. Besides, CS-g-CA caused the accumulation of intracellular ROS, repressed the production of the quorum-sensing signaling molecules and c-di-GMP. CA exhibited significant anti-biofilm efficacy against *Klebsiella pneumoniae* by lowering the expression of MrkD and TreC but not of LuxS, the synthesase for type II autoinducer (AI-2) of quorum-sensing system. MrkD (Type 3 fimbriae) and TreC (trehalose-6-phosphate hydrolase) are involved in biofilm formation in *K. pneumoniae*, and MrkD enables surface adhesion [53], and TreC plays a positive role in biofilm formation, initial adhesion, surface colonization, and dispersion [54]. It can be seen that the anti-biofilm mechanism of CA is intricate and differs across the bacterial types. The underlying anti-biofilm mechanism of CA against *V. parahemolyticus* should be uncovered in further work.

### 3.4. Changes in Bacterial Colonies of Salmon After Treatment with CA

**Figure 6:**
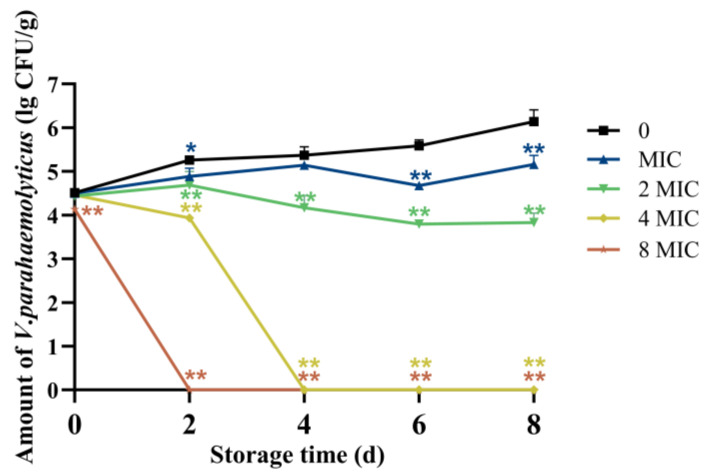
Effect of chlorogenic acid at different concentrations against *V. parahaemolyticus* in salmon. Each value represents the average of three independent measurements. The bar represents the standard deviation (n = 3), * *p* < 0.05, ** *p* < 0.01.

Raw salmon fillet is a popular ready-to-eat seafood worldwide because of its exceptional taste, nutritional value, and health benefits. However, contamination with *V. parahaemolyticus* presents substantial health hazards to consumers. To evaluate the antibacterial potential of CA in a food matrix, raw salmon fillets were used as a food model, with an initial *V. parahaemolyticus* contamination level of approximately 4.46 log CFU/g. During the storage period, the total *Vibrio* populations in the control groups with no CA treatment increased along with the prolonged storage time, reaching 6.14 lg CFU/g on the 8th day. In the 8 MIC and 4 MIC treatment groups, *V. parahaemolyticus* was completely killed on the 2nd and 4th days, respectively. While in the non-CA treatment group, the total *V. parahaemolyticus* count reached 5.26 and 5.37 lg CFU/g on the 2nd and 4th days, respectively. *V. parahaemolyticus* clearance rates on the 8th day following CA therapy were 100% for 8 MIC, 100% for 4 MIC, and 13.74% for 2 MIC, respectively (Figure 6). These results demonstrated that CA effectively removes and inactivates *V. parahaemolyticus* on the surface of RSF, with higher concentrations achieving complete eradication.

## 4. Conclusions

Chlorogenic acid displayed a good antibacterial effect against *V. parahaemolyticus*, with a minimum inhibitory concentration of 6 mg/mL. CA can affect the integrity and permeability of the cell membrane and cell wall, leading to the leakage of large and small molecules inside the cell, thereby achieving antibacterial effects. At sub-inhibitory concentrations, CA repressed the production of the virulence factors, including motility, extracellular polysaccharides, and biofilm of *V. parahaemolyticus*. Furthermore, CA exhibited protective potential against *V. parahaemolyticus* in salmon; however, further studies are needed to evaluate its safety and potential applications as a food antibacterial agent.

## Data Availability

The original contributions presented in this study are included in the article. Further inquiries can be directed to the corresponding author.
